# Analysis of variable metabolites in preterm infants with bronchopulmonary dysplasia: a systematic review and meta-analysis

**DOI:** 10.1186/s13052-024-01812-9

**Published:** 2024-11-14

**Authors:** Yanping Guo, Ying Liu, Ruolin Zhang, Songzhou Xu, Xin Guo, Zhangbin Yu, Guobing Chen

**Affiliations:** 1 Department of Pediatrics, Peking University Shenzhen Hospital, Shenzhen, China; 2 Department of Pediatrics, Division of Neonatology, Nanshan Maternity & Child Healthcare Hospital, Shenzhen, China; 3 Division of Neonatology, Longgang District Maternity & Child Healthcare Hospital of Shenzhen City (Longgang Maternity and Child Institute of Shantou University Medical College), Shenzhen, China; 4 Department of Pediatrics, Division of Neonatology, Shenzhen People’s Hospital, The Second Clinical Medical College, Jinan University, The First Affiliated Hospital, Southern University of Science and Technology, Shenzhen, China

**Keywords:** Bronchopulmonary dysplasia, Variable metabolites, Metabolomics, Meta-analysis

## Abstract

Numerous studies have attempted to identify potential biomarkers for early detection of bronchopulmonary dysplasia (BPD) in preterm infants using metabolomics techniques. However, the presence of consistent evidence remains elusive. Our study aimed to conduct a systematic review and meta-analysis to identify differences in small-molecule metabolites between BPD and non-BPD preterm infants. Through meticulous screening of numerous samples, we identified promising candidates, providing valuable insights for future research. We searched PubMed, the Cochrane Library, Embase, Web of Science, China National Knowledge Internet, Wan-fang database, Chinese Science and Technique Journal Database and Chinese Biomedical Literature Database from inception until January 16, 2024. Studies were comprehensively reviewed against inclusion criteria. We included case-control studies and adhered to Preferred Reporting Items for Systematic Reviews and Meta-Analysis guidelines. Study quality was assessed with the Newcastle-Ottawa scale. We compared the changes in metabolite levels between the BPD and non-BPD preterm infants. A meta-analysis was conducted on targeted metabolomics research data based on the strategy of standardized mean differences (MD) and 95% confidence intervals (CI).Fifteen studies (1357 participants) were included. These clinical-based metabolomics studies clarified 110 differential metabolites between BPD and non-BPD preterm infants. The meta-analysis revealed higher glutamate concentration in the BPD group compared to the non-BPD group (MD = 1, 95% CI 0.59 to 1.41, *p* < 0.00001). Amino acids were identified as the key metabolites distinguishing preterm infants with and without BPD, with glutamate potentially serving as a BPD predictor in this population.

**Supplementary Information:**

The online version contains supplementary material available at 10.1186/s13052-024-01812-9.

## Background

Bronchopulmonary dysplasia (BPD) is one of the most common and significant complications of premature birth. However, despite significant advancements in healthcare [1], the incidence of BPD remains high, with no noticeable decrease observed [2, 3] BPD not only prolongs hospital stays and increases healthcare expenses for premature infants but also leads to adverse respiratory and neurological outcomes, with the potential for fatal consequences [4–8]. Due to the diverse clinical symptoms and the complexity influenced by multiple factors, there is currently a lack of objective standards to accurately predict the mortality and incidence rates of BPD [9–12]. Therefore, the search for biomarkers capable of early predicting BPD in preterm infants is the current focus and challenge of research.

A growing body of clinical and experimental evidence indicates that insufficient maternal nutrition during the perinatal period and early-life nutritional deficiencies are significant risk factors for BPD and impaired lung function in premature infants [13–16]. Research also suggests that metabolic reprogramming emerges as a significant characteristic at the onset of BPD, primarily characterized by abnormalities in glucose, lipid, amino acid, and other metabolic pathways [17]. Thus, utilizing metabolomic techniques to identify biomarkers in early-life biological samples from preterm infants (such as umbilical cord blood, urine, tracheal aspirates, or blood) may rapidly detect potential metabolic disturbances and identify unique metabolites for early prediction of BPD [18–20]. Early identification of newborns at risk of developing BPD, coupled with timely targeted interventions, can assist in preventing and reducing the severity of the condition.

Metabolomics entails a comprehensive and systematic analysis of a variety of small molecule metabolites through high-throughput techniques. It reveals the distinctive metabolic characteristics of organisms, thus providing a means to discern physiological or pathological states [21]. Currently, metabolomics is widely utilized in the field of neonatology, helping in the early identification and diagnosis of various diseases [22, 23]. While scholars explore BPD metabolomics, yet consistent conclusions on metabolic differences in premature infants with and without BPD are elusive. Enhanced understanding enhances disease comprehension.

Hence, we conducted an exhaustive systematic review and meta-analysis of small-molecule metabolites in premature infants with and without BPD. This endeavor establishes essential groundwork for future metabolomic investigations in samples of premature infants with BPD.

## Methods

Our systematic review and meta-analysis adhered to the standard criteria Preferred Reporting Items for Systematic Reviews and Meta-Analysis (PRISMA) [24]. This research protocol has been registered in the International Prospective Register of Systematic Reviews (PROSPERO CRD42024504179).

### Data sources and search strategy

PubMed, the Cochrane Library, Embase, Web of Science, China National Knowledge Internet (CNKI), Wan-fang database, Chinese Science and Technique Journal Database (VIP) and Chinese Biomedical Literature Database (CBM) were systematically searched for relevant articles published from inception up to January 16, 2024, without restrictions on countries or article type. Our search strategy combined interventions (Metabolomics) with diseases (BPD) in preterm infants. The detailed search strategy is outlined in Supplementary Table S1. Additionally, a comprehensive manual search of the reference lists of all selected articles was conducted to ensure that no relevant studies were inadvertently missed during the initial search process. The screening of bibliographies was conducted independently to further enhance the comprehensiveness of the search.

### Eligibility criteria

All included studies satisfied the following inclusion criteria: (1) clinical study of BPD preterm infants (cohort study, case-cohort study, case‐control or clinical trial); (2) the study must include BPD group and control group; (3) incorporating metabolomics studies on human biological samples (urine, blood, tracheal aspirates, etc.); (4) studies reporting on differential small molecule metabolites between preterm infants with and without BPD.

The exclusion criteria were as follows: (1) duplicate publications; (2) unavailability of full-text; (3) non-original papers, such as meeting abstracts, letters and reviews; (4) insufficient information.

### Study selection

All identified records were downloaded into EndNote X9, and duplicates were subsequently removed. Two independent researchers screened the studies based on their titles and abstracts, respectively. Subsequently, the studies meeting our criteria underwent full-text screening for further evaluation. Any discrepancies were resolved through discussion with a third researcher until a consensus was reached within the team.

### Data extraction

Two independent researchers extracted information from eligible articles, including the first name of the author, year of publication, study design type, number of BPD and control, the recruitment area or country, diagnostic criteria for BPD, gestational age (GA), metabolomics technique, biological sample, the name of different metabolites, the variation trend, and the concentration of metabolites and associated metabolic pathways. To convert the median and quartiles to mean and standard deviation (SD), we first assessed skewness and then applied a novel piecewise function based on the sample size [25, 26]. In cases where two experimental groups were reported but matched with a single control group, the mean and standard deviation of the experimental groups were combined using the formula outlined in the Cochrane Manual (Supplementary Table S2). Classify different metabolites according to amino acid, lipid, carbohydrate, and other metabolites.

### Risk of bias assessment

We assessed the quality of the case-control studies using the Newcastle-Ottawa Scale (NOS), which evaluates aspects such as random sample selection, comparability of cases and controls, and exposure [27]. Each criterion related to selection and exposure could receive up to one point, while comparability could receive up to two points. Two independent researchers conducted the assessment, with any disagreements resolved through arbitration by a third researcher.

### Statistical analysis

We used Review Manager (Version 5.4.1) to conduct a meta-analysis based on clinical metabolomics. A qualitative analysis was conducted for various metabolites by counting their frequency across the included studies. When two or more studies reported the concentrations of the same metabolites, a meta-analysis was carried out using the mean difference (MD) with 95% confidence intervals (95% CI). A random-effects model was applied in cases of high heterogeneity (*I*^*2*^ > 50%), while a fixed-effects model was utilized otherwise. Sensitivity analyses were performed to evaluate the potential impact of biases by systematically removing one study at a time.

## Results

### Literature search and study selection

**Fig. 1:**
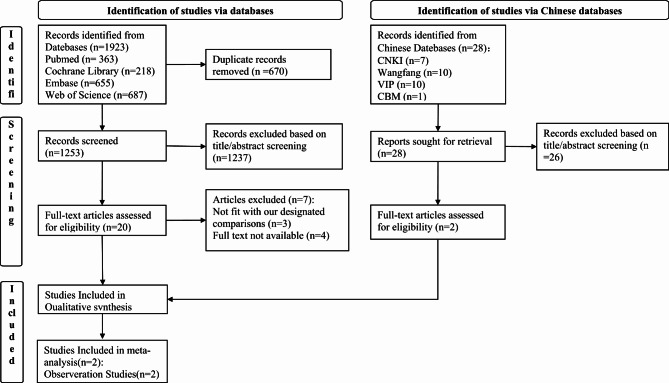
Flow chart for the selection of studies

The literature search and study selection are shown in Fig. 1. Among the 1923 titles and abstracts, 670 duplicate records were deleted, and 1237 unrelated studies were removed. Of the remaining 20 studies, 3 were not eligible according to our criteria, 4 were excluded due to not access to full test. Ultimately, we included 15 studies [28–42] and 1357 participants (649 in BPD group and 708 in no BPD group).

### Characteristics of the included studies

All of the included studies were published between 2000 and 2024 and designed as case–control studies. Thirteen studies [28–32, 35–42] were reported in English and two studies [33, 34] in Chinese. The participants were recruited from United States, United Kingdom, Italy, Germany, Mexico and China. All participants in the study were born at a gestational age of 34 weeks or less. The studies were classified according to different sample types, including 4 urine samples [29, 31, 33, 42], 4 tracheal aspirate samples [28, 35, 39, 40], 3 dried blood spot samples [34, 37, 41], 1 amniotic fluid sample [30], 1 umbilical cord blood sample [32], 1 exhaled condensate sample [38], 1 breast milk and stool sample [36]. All the included studies measured metabolites mostly using liquid chromatography-mass spectrometry (LC-MS) or chromatography-mass spectrometry (GC-MS) techniques. The characteristics of each study are shown in Table 1.

**Table 1:** Characteristics of studies included in the systematic review and meta-analysis

Author (Year)	country	sample	Sample size (BPD/NO BPD)	GA(Weeks)	Metabolomics technique	NOS
López-Hernández(2024)	Mexico	Urinary	12/13	< 34	LC-MS/MS	6
Course(2023)	United Kingdom	exhaled breath condensate	34/110	≤ 34	GCTOF-MS	6
Wang(2022)	China	dried blood spots	45/40	< 33	MS/MS	7
Frazer(2022)	United States	Stool	45/27	< 28	LC-MS/MS	7
Ye(2022)	United States	dried blood spots	355/395	< 32	LC-MS/MS	6
Xu(2022)	China	tracheal aspirate	23/5	≤ 34	UPLC-TQ-MS	6
Piersigilli(2019)	United States	tracheal aspirate	44/24	< 30	LC-MS/MS	7
Li(2019)	China	dried blood spots	20/22	< 32	LC-MS/MS	6
Huang(2019)	China	Urinary	20/20	< 32	GC-MS	6
Hendricks-Muñoz(2018)	United States	tracheal aspirate	16/9	< 32	LC-MS/MS	6
La Frano(2018)	United States	Umbilical cord blood	10/10	< 32	GC-MS	6
Pintus(2018)	Italy	Urinary	7/11	< 28	1 H-NMR	5
Baraldi(2016)	Italy	Amniotic Fluid	10/11	21–28 of pregnancy	UPLC-TQ-MS	7
Fanos(2014)	Italy	Urinary	18/18	< 29	NMR	6
Rüdiger(2000)	Germany	tracheal aspirate	10/15	≤ 32	GC	7

Note: BPD, bronchopulmonary dysplasia; GA, gestational age; NOS, Newcastle–Ottawa Scale; GCTOF-MS, Gas Chromatography Time-of-Flight Mass Spectrometry; MS/MS, tandem mass spectrometry; LC-MS/MS, Liquid Chromatography Tandem Mass Spectrometry; GC-MS, Gas Chromatography–Flight Mass Spectrometry; 1 H-NMR, Hydro-Nuclear Magnetic Resonance Spectrometer; UPLC-TQ-MS, Ultra Performance Liquid Chromatography–Time of Flight–Mass Spectrometry; NMR, Nuclear Magnetic Resonance spectroscopy; GC, Gas Chromatography

From a total of 15 studies, we identified 110 differentially expressed small molecule metabolites. These included 36 metabolites from tracheal aspirate samples, 36 from urine samples, 25 from dried blood spot samples, 10 from exhaled condensate samples, 8 from amniotic fluid samples, 3 from umbilical cord blood samples, and 1 from stool samples (as shown in Table 2). Among these, 3 metabolites appeared more than twice and could be qualitatively synthesized. Only one metabolite had available concentration data from two investigations [34, 35]. Metabolite was measured in both tracheal aspirate and dried blood spot samples, with concentrations reported in µmol/L. For specific details, please refer to Supplementary Table S3.

**Table 2:** Qualitative synthesis results of differential small molecule metabolites between BPD and no BPD

	Differential small molecule metabolites name			
Sample	Patients predisposed to BPD		Patients not predisposed to BPD	
Concentration Trend	upward	downward	upward	downward
exhaled breath condensate	Maleimide, Octadecanol	Urea, Pyroglutamic acid, Valine, Triethanolamine, Histidine, Alanine, Ornithine, Serine		
Dried blood spot	three carnitines (C0, C2, and C6:1), glutamate, ornithine, phenylalanine, methionine, hydroxypalmitoylcarnitine	threonine, arginine, methionine, glutamine, glycine, proline, tryptophan, piperazine, Citrulline, alanine, glutamate, tyrosine, propionylcarnitine, free carnitine, acetylcarnitine, hydroxybutyrylcarnitine, and median-chain acylcarnitines (C5:C10)		
Stool		Acetic Acid		
tracheal aspirate	Histidine, glutamate, citrulline, glycine, isoleucine, Serine, acylcarnitines C16-OH, C14:1-OH, C10:1, C14:2-OH, C18:1-OH, C12:1, PCaaC24:0, PCaaC26:0, PCaaC38:5, PCaeC38:1, PCaeC36:3, PCaeC42:4, PCaeC44:5, lysoPCaC16:1, lysoPCaC28:1, lysoPCaC26:0, lysoPCaC14:0, lysoPCaC20:4, sn-glycerol 3-phosphoethanolamine, Sphingosine 1-phosphate, ceramides C14:0, ceramides C22:0, monohexosylceramide C18:1, monohexosylceramide C22:0, monohexosylceramide C26:0, sphingomyelin C18:0, sphingomyelin C20:0		polyunsaturated fatty acid, plasmalogens, Symmetric dimethylarginine	
Urinary	canine uric acid, thymine, alanine, betaine, lactate, taurine, trimethylamine-N-oxide, myoinositol, tyrosine, proline, Fumaric acid, 2-oxoisocaproic acid, 2-hydroxybutyric acid, acylcarnitines C0, acylcarnitines C2, acylcarnitines C4-OH, acylcarnitines C4, acylcarnitines C5, acylcarnitines C5:1DC	trehalose, tartaric acid, trimethylamine-N-oxides, lactate, glycine, gluconate, serotonin, 5-hydroxyl indoleacetic acid, indoxyl sulfates, allantoin, homocitrulline		
Umbilical cord blood	choline	phosphatidylcholines, sphingomyelins		
Amniotic Fluid	leucinic acid, 4-Hydroxy3-methylbenzoic acid, 2-hydroxy caprylic acid, 3-oxododecanoic acid, sulphated steroid	S-adenosylmethionine, aminoacid chains	3b,16a-Dihydroxyandrostenone sulfate	

Note: C0, Carnitine; C2, Acetylcarnitine; C6:1, Hexenoylcarnitine; C16-OH, Hydroxyhexadecanoylcarnitine; C14:1-OH, Hydroxytetradecenoylcarnitine; C10:1, Decenoylcarnitine; C14:2-OH, Hydroxytetradecadienylcarnitine; C18:1-OH, Hydroxyoctadecenoylcarnitine; C12:1, Dodecenoylcarnitine; PCaa, Phosphatidylcholine diacyl; PCae, Phosphatidylcholine acyl-alkyl; lysoPCa, Lysophosphatidylcholine acyl; C24:0, Lignoceroylcarnitine; C26:0, Cerotoylcarnitine; C38:5, Hexatriacontapentaenoylcarnitine; C38:1, Octatriacontenoylcarnitine; C36:3, triacylcarnitine; C42:4, tetraeicosatetraenoylcarnitine; C44:5, pentacosapentaenoylcarnitine; C16:1, hexadecenoylcarnitine; C28:1, octacosanoylcarnitine; C14:0, tetradecanoylcarnitine; C20:4, eicosatetraenoylcarnitine; C22:0, docosanoylcarnitine; C18:1, octadecenoylcarnitine; C4-OH, 4-hydroxybutyrylcarnitine; C4, butyrylcarnitine; C5, isovalerylcarnitine; C10, decanoylcarnitine; C5:1DC, glutarylcarnitine

### Assessment of risk of bias

The NOS scores assessed case-control studies, with studies scoring 5 stars or above deemed moderate to high quality. Overall, most studies scored 6 stars or above, indicating their quality. One study scored below 5 stars due to a weak case definition. All studies met meta-analysis requirements. Risk of bias is detailed in Table 1, with more in Supplementary Table S4.

### Primary outcomes

#### Qualitative synthesis amino acids

In the 15 studies, 110 differential small molecule metabolites were qualitatively synthesized by counting the frequency of change direction. Compared with no BPD, 67 increased, 39 decreased, and 12 showed direction conflicts; compared with BPD, 4 increased. Classified by sample type, 36 metabolites in tracheal aspirate samples increased; in exhaled breath condensate samples, 2 increased, 8 decreased; in dried blood spot samples, 8 increased, 17 decreased; in urine samples, 19 increased, 11 decreased, and 2 showed inconsistency; in amniotic fluid samples, 6 increased, 2 decreased; and in umbilical cord blood samples, 1 increased, 2 decreased. One metabolite decreased in stool samples. These diverse metabolites include amino acids, lipids, carbohydrates, and others (as shown in Table 2). Detailed information on the 110 differentially expressed small molecule metabolites is provided in Supplementary Table S5.

### Amino acid metabolite differences

Ten studies [29–31, 33–35, 37, 38, 41, 42] confirmed differences in amino acid and metabolite levels between BPD and non-BPD preterm infants. Fanos et al. [29] observed higher taurine and trimethylamine-N-oxide levels in urine of BPD infants born at 29 weeks, while Pintus et al. [31] reported elevated proline and betaine concentrations in urine of BPD infants born at < 28 weeks. Baraldi et al. [30] found lower s-adenosylmethionine and higher leucine concentrations in amniotic fluid of infants later developing BPD. Wang et al. [37] noted lower serine, arginine, histidine, and glutamine levels in BPD preterm infants’ dried blood spots. In a large sample study by Ye et al. [41], it was found that the concentrations of phenylalanine and methionine were elevated in the dry blood spots of infants with BPD compared to the non-BPD group, while the levels of citrulline, alanine, glutamate, and tyrosine were decreased. López-Hernández et al. [42] detected urine metabolites at 24 h postnatal and found higher concentrations of proline and tyrosine in the BPD group. Piersigilli et al. [35] observed higher arginine, glutamate, glutamine, glycine, and isoleucine levels in the tracheal aspirates of BPD infants. Additionally, two Chinese studies [33, 34] reported elevated glutamine and urea levels in dried blood spots of BPD infants and increased uric acid concentrations in their urine specimens. Course et al. [38] documented reduced valine, histidine, alanine, asparagine, and serine levels in exhaled breath condensate of BPD children aged 7–12 years.

### Lipid metabolite differences

Six studies [28, 30, 32, 38–40] highlighted differences in lipid metabolites between BPD and non-BPD preterm infants. Baraldi et al. [30] observed lower phosphatidylcholine levels in amniotic fluid samples from BPD infants. La Frano et al. [32] associated cord blood phosphatidylcholine levels with BPD occurrence and severity. Rüdiger et al. [28] detected higher unsaturated fatty acids in tracheal aspirate samples post-birth from non-BPD preterm infants. Xu et al. [40] found that within the first week postnatally, premature infants with BPD exhibited increased concentrations of sn-glycerol 3-phosphoethanolamine in tracheal aspirates, which positively correlated with the severity of BPD. Similarly, Hendricks-Muñoz et al. [39] observed elevated levels of sphingosine 1-phosphate and selective sphingoid bases in tracheal aspirates within the first week postnatally in premature infants with BPD. Additionally, Course et al. [38] reported elevated glycerophospholipids in breath condensate of adolescents with BPD.

### Carbohydrate metabolite differences

Current research found glucose metabolism issues in BPD preterm infants. Fanos et al. [29] noted low urinary gluconic acid in BPD infants at birth.

### Other metabolites differences

Organic acids: López-Hernández et al. [42] found that premature infants with BPD exhibited increased concentrations of organic acids such as fumaric acid, 2-oxoisocaproic acid, and 2-hydroxybutyric acid in urine within 24 h postnatally. Fanos et al. [29] found high urinary lactate levels in BPD infants at birth, while Pintus et al. [31] observed decreased urinary lactate levels on day 7 post-birth in BPD infants. In one study [36], lower fecal acetic acid levels were seen in BPD preterm infants.

Acylcarnitines: Concentrations of Hydroxypalmitoylcarnitine [41], acylcarnitines, including hydroxyhexadecanoylcarnitine (C16-OH) [35], hydroxyoctadecenoylcarnitine (C18:1-OH) [35], Carnitine (C0) [42], acetylcarnitine (C2) [42], 4-hydroxybutyrylcarnitine (C4-OH) [42], butyrylcarnitine(C4) [42], isovalerylcarnitine(C5) [42], and glutarylcarnitine (C5:1DC) [42], as well as three other carnitines [C0, C2, and hexenoylcarnitine (C6:1)] [37], were elevated in BPD preterm infants. Propionylcarnitine, free carnitine, acetylcarnitine, hydroxybutyrylcarnitine, and most median-chain acylcarnitines [C5: decanoylcarnitine(C10)] were down-regulated in BPD babies over the early days of life [41].

Other compounds: Sulfated steroids [30] and thymidine [33] exhibited increased concentrations in BPD preterm infants, whereas concentrations of trehalose and tartaric acid [33] decreased. Serotonin, 5-hydroxyl indoleacetic acid, indoxyl sulfate, and other amino acid derivatives (allantoin and homocitrulline) were found in lower levels in the BPD group [42].

### Distinct metabolic pathways

Two studies [37, 38] delineated distinct metabolic pathways in preterm infants with and without BPD, detailed in Table 3. These pathways include alanine, aspartate, and glutamate metabolism; cysteine and methionine metabolism; the urea cycle; glutathione metabolism; methionine metabolism; as well as arginine and proline metabolism.

**Table 3:** Associated metabolic pathways involved in BPD

Study	Pathways	Analysis methods
Course(2023)	Urea cycle	Small Molecule Pathways Database (SMPDB), which is based on the Human Metabolome Database (HMDB)
	Glutathione metabolism	
	Methylhistidine metabolism	
	Arginine and proline metabolism	
Wang(2022)	Alanine, aspartate, and glutamate metabolism	MetaboAnalyst 4.0 software
	Cysteine and methionine metabolism	

### Meta-analysis for metabolites

**Fig. 2:**
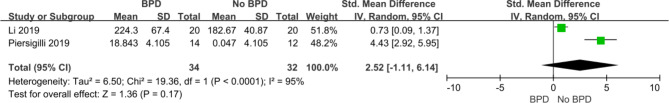
Forest plot of the concentration of glutamine in BPD and No BPD (µmol/L). Note: The green boxes represent the point estimates for each study, and the black boxes represent the combined values of the study results

If two or more studies provide a metabolite’s concentration with mean and standard deviation, it’s included in the meta-analysis. Due to limited consistency, only glutamine was analyzed [34, 35]. Results show higher glutamine levels in the BPD group than No BPD (MD = 1, 95% CI 0.59 to 1.41, *p* < 0.00001) (Fig. 2). With low heterogeneity, a fixed-effect model was used.

## Discussion

This systematic review and meta-analysis the first identified numerous metabolites associated with BPD infants. Nevertheless, only one differential small molecule metabolite was identified in the meta-analysis, showing increased glutamate concentrations in tracheal aspirates and dried blood spots of BPD compared to non-BPD infants. Despite some heterogeneity in the meta-analysis results, the estimated effects from these two studies were relatively consistent. Glutamate may be a potential candidate biomarker worthy of further exploration. Moreover, this systematic review also detected several metabolic pathways associated with preterm BPD, primarily involving amino acid metabolism.

The disruption of amino acid metabolism in BPD preterm infants is primarily as-sociated with inflammation and oxidative stress, although specific patterns are still debated. Trimethylamine-N-oxide maintains the stability of biological membranes, while taurine helps regulate cellular osmotic balance, both of which may serve as potential indicators of fetal health [43]. Betaine, functioning as a non-essential amino acid and quaternary ammonium compound in various biochemical processes, has been shown to have protective effects against lung injury [44, 45]. Fanos et al. [25] observed higher levels of taurine and trimethylamine-N-oxide in the urine of BPD infants, while Pintus et al. [27] observed elevated levels of alanine and betaine in the urine of BPD infants, along with decreased concentrations of trimethylamine-N-oxide and glycine. Variations in the timing of sample collection may contribute to discrepancies in trimethylamine-N-oxide levels among urine metabolites in the two studies. Premature infants commonly experience hypoxia within the first 24 to 36 h after birth, prompting glycolysis activation and subsequent release of protective substances to maintain membrane stability, resulting in increased levels of taurine and trimethylamine-N-oxide shortly after birth. Additionally, increased resting energy expenditure in BPD infants may stimulate the glucose-alanine cycle, leading to elevated levels of ala-nine. Glutamine, arginine, and glycine have been shown to exhibit protective effects in lung injury models through anti-inflammatory mechanisms [46, 47]. Methionine serves as a crucial cellular antioxidant, which can be converted to cysteine to replenish intracellular glutamine stores, while citrulline acts as a precursor of arginine.

Lipidomics suggests a potential correlation between disrupted lipid metabolism and the onset of BPD. Phospholipids constitute the primary components of biological membranes. La Frano et al. [28] identified a notable correlation between phosphatidylcholine levels in umbilical cord blood and the onset of BPD, demonstrating an in-verse relationship with disease severity. Acetyl carnitine is released during the process of fatty acid beta-oxidation. Supplementing neonatal mice with L-carnitine one week after exposure to high oxygen levels can alleviate oxygen-induced cell apoptosis and lung injury [48]. Carraro et al. [49] found that from infancy to adolescence, individuals with BPD continued to manifest alterations in the lipid profile of their exhaled condensate, indicating the potential persistence of abnormal lipid metabolism beyond infancy.

Our study revealed the association between organic acids, particularly lactic acid, and BPD. Lactic acid, a byproduct of glycolysis, contributes to this association. Premature infants with BPD are born in a relatively hypoxic state, thereby stimulating heightened glycolytic activity. Subsequently, under hyperoxic conditions, glycolysis is attenuated, leading to impaired function of complexes I and II in pulmonary mitochondria, thus restricting energy production [50].

### Strengths and limitations

Through a systematic review and meta-analysis, we meticulously analyzed differences in small-molecule metabolites between BPD and non-BPD preterm infants. Our comprehensive literature search across major English and Chinese databases minimized the likelihood of overlooking crucial reports. This study represents the first qualitative synthesis to assess the correlation between various metabolites and the development of BPD in preterm infants. Additionally, we provided a thorough description of pertinent metabolic pathways, enhancing the understanding of BPD pathogenesis. Despite significant heterogeneity in most findings, the studies included in our analysis demonstrated high quality.

Despite the innovation of our study, it is crucial to recognize several limitations. Firstly, Inconsistent reporting of results and unavailability of raw data in most of the included studies resulted in the meta-analysis being limited to one metabolite. Secondly, heterogeneity arose from variations in gestational age among preterm infants, types of biological samples collected, and diverse methods of metabolite detection. Thirdly, the identification of different metabolites relied solely on primary literature and lacks original data, and we were unable to evaluate the diagnostic value of candidate metabolic biomarkers through receiver operating characteristic curve (ROC) analysis. However, due to the limited number of articles included in the meta-analysis, we did not conduct subgroup analysis to account for these effects. Finally, as our study comprised retrospective case-control studies, our findings can only offer insights into identifying predictive metabolites.

## Conclusions

Amino acids, particularly glutamate, have been identified as key metabolites that differentiate preterm infants with BPD from those without. These findings indicate that metabolomic profiling can play a crucial role in the early prediction and diagnosis of BPD, potentially enabling more targeted interventions and improving outcomes for preterm infants. Future research should aim to validate these biomarkers and investigate their mechanistic roles in the development of BPD.

## Electronic supplementary material

Below is the link to the electronic supplementary material.

Supplementary Material 1

Supplementary Material 2

## Data Availability

The data underlying this article are available within the article and its online supplementary material.

## Abbreviations

BPD: Bronchopulmonary dysplasia
GA: Gestational age
SD: Standard deviation
MD: Mean difference
CI: Confidence interval
I^2^: Inconsistency index
C0: Carnitine
C2: Acetylcarnitine
C5: Isovalerylcarnitine
