# Supplementary material for: Analysis of variable metabolites in preterm infants with bronchopulmonary dysplasia: a systematic review and meta-analysis
Source: Ital J Pediatr. 2024 Nov 14;50:246. doi: 10.1186/s13052-024-01812-9 (PMC11566045; doi:10.1186/s13052-024-01812-9)
Supplement: Supplementary file 2 — Supplementary Material 2 [file 13052_2024_1812_MOESM2_ESM.pdf]

Supplementary Materials

Tables

Table S1. Search strategies 2
Table S1.1. Pubmed search strategy 2
Table S1.2. Cochrane Central Register of Controlled Trials search strategy 2
Table S1.3. Embase search strategy 2
Table S1.4. Web of Science search strategy 3
Table S1.5. Chinese databases search strategy 3
Table S2. Formula 1 for combining mean and SD 4
Table S3. The characteristics for each differential metabolite 4
Table S4. The NOS assessment scale for every study 4
Table S5. Frequency of 110 differential small molecule metabolites 6
Table S5.1. Frequency of 106 differential small molecule metabolites of BPD 6
Table S5.2. Frequency of 4 differential small molecule metabolites of No BPD 9

Record of database search strategies - run 16/01/24

Table S1.1. Pubmed search strategy

#	Searches	Results
# 1	("infant, newborn"[Mesh Terms] OR "newborn*"[Title/Abstract] OR "new born"[Title/Abstract] OR "new borns" [Title/Abstract] OR "newly born"[Title/Abstract] OR "baby*"[Title/Abstract] OR "babies*"[Title/Abstract] OR "premature"[Title/Abstract] OR "prematurity"[Title/Abstract] OR "preterm"[Title/Abstract] OR "pre term"[Title/Abstract] OR "low birth weight'[Title/Abstract] OR "low birthweight"[Title/Abstract] OR "VLBW"[Title/Abstract] OR "LBW"[Title/Abstract] OR "infan*"[Title/Abstract]OR "neonat*"[Title/Abstract])	1335, 133
# 2	("Bronchopulmonary Dysplasia"[Mesh] OR "bronchopulmonary dysplasia"[Title/Abstract] OR "chronic lung disease"[Title/Abstract] OR "bronchopulmonary disease"[Title/Abstract] OR "BPD"[Title/Abstract])	25,649
# 3	((((metabolomics[Mesh Terms])OR(metabolomics[Title/Abstract]))OR (metabolomics[Title/Abstract]))OR (metabolomics[Title/Abstract]))OR (metabol*[Title/Abstract])	1,587,244
# 4	#1 AND #2 AND #3	363

Table S1.2. Cochrane search strategy

#	Searches	Results
# 1	Melabolomics	2005
# 2	(metabolomics):ti,ab,kw OR (metabonomics):ti,ab,kw OR (metabonomic):ti,ab,kw OR (metabol"):ti,ab,kw	157118
# 3	#1 OR #2	157151
# 4	(bronchopulmonary dysplasia): ti,ab,kw OR (chronic lung disease): ti,ab,kw OR (bronchopulmonary disease): ti,ab,kw OR (BPD): ti,ab,kw	21032
# 5	Bronchopulmonary Dysplasia	2076
# 6	#4 OR #5	21218
# 7	infant, newborn	26012
# 8	(newborn*):ti,ab,kw OR (premature):ti,ab,kw OR (prematurity):ti,ab,kw OR (preterm):ti,ab,kw OR low birth weicht)):ti,ab,kw	58433
# 9	#7 OR #8	58873
# 10	#3 AND #6 AND #9	216

Table S1.3. Embase search strategy

#	Searches	Results
# 1	'metabolomics'/exp OR metabolomics OR metabolomics:ab,ti OR metabonomics:ab,ti OR metabonomic:ab.ti OR metabol*:ab.ti	1908186
# 2	'bronchopulmonary dysplasia'/exp OR 'bronchopulmonary dysplasia' OR (bronchopulmonary AND ("dysplasia'/exp OR dysplasia)) OR 'bronchopulmonary dysplasia':ab,ti OR 'chronic lung disease':ab,ti OR 'bronchopulmonary disease':ab,ti OR bpd:ab,ti	40771
# 3	'infant, newborn'/exp OR 'infant, newborn' OR ('infant,'/exp OR infant,) AND ('newborn'/exp OR newborn)) OR newborn*:ab,ti OR 'new born': ab,ti OR 'newborns': ab,ti OR 'newly born': ab,ti OR baby*: ab.ti OR babies*: ab.ti OR premature: ab.ti OR prematurity;ab,ti OR preterm: ab.ti OR 'pre term': ab.ti OR "low birthweight':ab,ti OR "low birthweight':ab,ti OR vlbw:ab,ti OR lbw:ab,ti OR infan*:ab,ti OR neonat*:ab,ti	1571399
# 4	#1 AND #2 AND #3	655

Table S1.4. Web of Science search strategy

#	Searches	Results
# 1	metabolomics (Topic)	55008
# 2	metabonomics (Topic)	4894
# 3	metabonomic (Topic)	1943
# 4	metabol*(Topic)	1950819
# 5	#1 OR #2 OR #3 OR #4	1951356
# 6	TS=(Bronchopulmonary Dysplasia)	12746
# 7	TS=(chronic lung disease)	66459
# 8	TS=(bronchopulmonary disease)	8432
#9	TS=(BPD)	13754
#10	#6 OR #7 OR #8 OR #9	87689
#11	TS=(infant, newborn)	66554
#12	TS=(newborn*)	180853
#13	TS=(new born)	131870
#14	TS=(new borns)	985
#15	TS=(newly born)	13575
#16	TS=(baby*)	56704
#17	TS=(babies*)	37824
#18	TS=(premature)	165121
#19	TS=(prematurity)	29532
#20	TS=(preterm)	123690
#21	TS=(pre term)	142536
#22	TS=(low birth weight)	75123
#23	TS=(low birthweight)	11765
#24	TS=(VLBW)	4897
#25	TS=(LBW)	5363
#26	TS=(infan*)	576847
#27	TS=(neonat*)	328895
#28	#11 OR #12 OR #13 OR #14 OR #15 OR #16 OR #17 OR #18 OR #19 OR #20 OR #21 OR #22 OR #23 OR #24 OR #25 OR #26 OR #27	1381173
#29	#5 AND #10 AND #28	687

Table S1.5. **Chinese datebases search strategy**

China National Knowledge Infrastructure (CNKI)	
(主题=早产 + 早产儿 + 低出生体重儿 + VLB)<br>AND<br>(主题=支气管肺发育不良+慢性肺疾病)<br>AND<br>(主题=代谢组学+代谢物+生物标志物)	*7*
**Wanfang Database (Wangfang)**	
(题名或关键词=早产 OR早产儿OR 低出生体重儿 OR VLB)<br>AND<br>(题名或关键词=支气管肺发育不良OR慢性肺疾病)<br>AND<br>(题名或关键词=代谢组学 OR代谢物 OR生物标志物)	*10*
**China Science and Technology Journal Database (VIP)**	
(题名或关键词=早产 + 早产儿 + 低出生体重儿 + VLB)<br>AND	*10*

(题名或关键词=支气管肺发育不良 + 慢性肺疾病)<br>AND<br>(题名或关键词=代谢组学 + 代谢物 + 生物标志物)	
**China Biology Medicine disc (CBM)**	
("早产"[标题:智能] OR "早产儿"[标题:智能] OR "低出生体重儿"[标题:智能] OR "VLB"[标题:智能])<br>AND<br>("支气管肺发育不良"[标题:智能] OR "慢性肺疾病"[标题:智能])<br>AND<br>("代谢组学"[摘要:智能] OR "代谢物"[摘要:智能] OR "生物标志物"[摘要:智能])	*1*

Table S2 Formula 1 for combining mean and SD

	Group 1	Group 2	Combined Group
Sample Size	N_1	N_2	$N_1 + N_2$
Mean	M_1	M_2	$\frac{N_1 M_1 + N_2 M_2}{N_1 + N_2}$
SD	SD_1	SD_2	$\sqrt{\frac{(N_1 - 1) SD_1^2 + (N_2 - 1) SD_2^2 + \frac{N_1 N_2}{N_1 + N_2}(M_1^2 + M_2^2 - 2M_1 M_2)}{N_1 + N_2 - 1}}$

Table S3 The characteristic for each differential metabolites

Metabolite name	Year	Author	Mass spectrometry techniques	Sample	Original Unit	Data soure
glutamate	2019	Piersigilli	LC-MS/MS	tracheal aspirate	μmol / L	Digit
glutamate	2019	Li	MS-MS	Dried blood spot	μmol / L	Digit

Table S4 The NOS assessment scale for every study

Study		Selection				Comparability	Exposure			scores
Year	Author	S1	S2	S3	S4	C1	E1	E2	E3	
**2024**	**López-Hernández**	A*	A*	B	A*	A*	A*	A*	B	6
**2023**	**Course**	A*	A*	B	A*	A*	A*	A*	B	6
**2022**	**Wang**	A*	A*	B	A*	A**	A*	A*	B	7
**2022**	**Frazer**	A*	A*	B	A*	A**	A*	A*	B	7
**2022**	**Ye**	A*	A*	B	A*	A*	A*	A*	B	6
**2022**	**Xu**	A*	A*	B	A*	A*	A*	A*	B	6
**2019**	**Piersigilli**	A*	A*	B	A*	A**	A*	A*	B	7
**2019**	**Li**	A*	A*	B	A*	A*	A*	A*	B	6
**2019**	**Huang**	A*	A*	B	A*	A*	A*	A*	B	6
**2018**	**Hendricks-Muñoz**	A*	A*	B	A*	A*	A*	A*	B	6

**2018**	**La Frano**	A*	A*	B	A*	A*	A*	A*	B	6
**2018**	**Pintus**	B	A*	B	A*	A*	A*	A*	B	5
**2016**	**Baraldi**	A*	A*	B	A*	A**	A*	A*	B	7
**2014**	**Fanos**	A*	A*	B	A*	A*	A*	A*	B	6
**2000**	**Rüdiger**	A*	A*	A	A*	A*	A*	A*	B	7

Note:
S1: Is the case definition adequate?
S2: Representativeness of the cases.
S3: Selection of Controls.
S4: Definition of Controls.
C1: Comparability of cases and controls on the basis of the design or analysis.
E1: Ascertainment of exposure.
E2: Same method of ascertainment for cases and controls.
E3: Non-Response rate.
* and ** mean score.

Table S5.1. Frequency of 106 differential small molecule metabolites of BPD

Decreased (patients predisposed to BPD)			Increased (patients predisposed to BPD)			Opposite trend			
NO.	Metabolite name	Research frequency	NO.	Metabolite name	Research frequency	NO.	Metabolite name	Increased frequency	Decreased frequency
1	Glycine	2	1	Glutamate	2	1	Glycine	1	2
2	Alanine	2	2	Ornithine	1	2	Histidine	1	1
3	Tryptophan	1	3	Histidine	1	3	Alanine	1	1
4	Threonine	1	4	Citrulline	1	4	Ornithine	1	1
5	Arginine	1	5	Glycine	1	5	Serine	1	1
6	Methionine	1	6	Isoleucine	1	6	Trimethylamine-N-oxides	1	1
7	Glutamine	1	7	Serine	1	7	Lactate	1	1
8	piperazine	1	8	canine uric acid	1	8	Methionine	1	1
9	Pyroglutamic acid	1	9	Leucine	1	9	Citrulline	1	1
10	Valine	1	10	Alanine	1	10	Glutamate	2	1
11	Histidine	1	11	Betaine	1	11	Tyrosine	1	1
12	Proline	1S	12	Thymine	1	12	Proline	1	1
13	Ornithine	1	13	Lactate	1				
14	Serine	1	14	Taurine	1				
15	S-adenosylmethionine	1	15	Trimethylamine-N-oxides	1				
16	Aminoacid chains	1	16	Myoinositol	1				
17	Urea	1	17	Maleimide	1				
18	Triethanolamine	1	18	Octadecanol	1				
19	Trimethylamine-N-oxides	1	19	Choline	1				
20	Lactate	1	20	Acylcarnitine C16-OH	1				
21	Phosphatidylcholines	1	21	Acylcarnitine C18:1-OH	1				

22	Tartaric acid	1	22	Carnitine C0	1				
23	Gluconate	1	23	Carnitine C2	1				
24	Acetic Acid	1	24	Carnitine C6:1	1				
25	Phosphatidylcholines	1	25	4-Hydroxy3-methylbenzoic acid	1				
26	Sphingomyelins	1	26	2-hydroxy caprylic acid	1				
27	Citrulline	1	27	3-oxododecanoic acid	1				
28	Glutamate	1	28	Sulphated steroid	1				
29	Tyrosine	1	29	C14:1-OH	1				
30	Propionylcarnitine	1	30	C10:1	1				
31	Free carnitine	1	31	C14:2-OH	1				
32	Acetylcarnitine	1	32	PC aa C24:0	1				
33	Hydroxybutyrylcarnitine	1	33	lysoPC a C28:1	1				
34	Median-chain Acylcarnitines (C5:C10)	1	34	C12:1	1				
35	Serotonin	1	35	lysoPC a C26:0	1				
36	5-hydroxyl indoleacetic acid	1	36	lysoPC a C14:0	1				
37	Indoxyl sulfates	1	37	PC aa C26:0	1				
38	Allantoin	1	38	PC ae C38:1	1				
39	Homocitrulline	1	39	PC ae C36:3	1				
			40	PC ae C42:4	1				
			41	lysoPC a C16:1	1				
			42	PC aa C38:5	1				
			43	lysoPC a C20:4	1				
			44	PC ae C44:5	1				

			45	Phenylalanine	1				
			46	Methionine	1				
			47	Tyrosine	1				
			48	Proline	1				
			49	Sn-glycerol 3-phosphoethanolamine	1				
			50	Hydroxypalmitoylcarnitine	1				
			51	Sphingosine 1-phosphate	1				
			52	Ceramides C14:0	1				
			53	Ceramides C22:0	1				
			54	Monohexosylceramide C18:1	1				
			55	Monohexosylceramide C22:0	1				
			56	Monohexosylceramide C26:0	1				
			57	Sphingomyelin C18:0	1				
			58	Sphingomyelin C20:0	1				
			59	Fumaric acid	1				
			60	2-oxoisocaproic acid	1				
			61	2-hydroxybutyric acid	1				
			62	Acylcarnitines C0	1				
			63	Acylcarnitines C2	1				
			64	Acylcarnitines C4-OH	1				
			65	Acylcarnitines C4	1				
			66	Acylcarnitines C5	1				
			67	Acylcarnitines C5:1DC	1				

Table S5.2 Frequency of 4 differential small molecule metabolites of No BPD

Decreased (patients no predisposed to BPD)			Increased (patients no predisposed to BPD)			Opposite trend			
NO.	Metabolite name	Research frequency	NO.	Metabolite name	Research frequency	NO.	Metabolite name	Increased frequency	Decreased frequency
			1	3b,16a-Dihydroxyandrostenone sulfate	1				
			2	polyunsaturated fatty acid	1				
			3	plasmalogens	1				
			4	Symmetric dimethylarginine	1				
