# Supplementary material for: Analysis of variable metabolites in preterm infants with bronchopulmonary dysplasia: a systematic review and meta-analysis
Source: Ital J Pediatr. 2024 Nov 14;50:246. doi: 10.1186/s13052-024-01812-9 (PMC11566045; doi:10.1186/s13052-024-01812-9)
Supplement: Supplementary file 1 — Supplementary Material 1 [file 13052_2024_1812_MOESM1_ESM.pdf]

Yanping Guo
Department of Pediatrics, Peking University Shenzhen Hospital, Shenzhen 518036, China
guoyanping1223@163.com
April 12, 2024.

Editor-in-Chief
Italian Journal of Pediatrics

Dear Editor-in-Chief,

I am pleased to submit our manuscript entitled " Analysis of Variable Metabolites in Preterm Infants with Bronchopulmonary Dysplasia： A Systematic Review and Meta-Analysis " for consideration of publication in Italian Journal of Pediatrics.

A growing body of clinical and experimental evidence indicates that insufficient maternal nutrition during the perinatal period and early-life nutritional deficiencies are significant risk factors for BPD and impaired lung function in premature infants. Research also suggests that metabolic reprogramming emerges as a significant characteristic at the onset of BPD, primarily characterized by abnormalities in glucose, lipid, amino acid, and other metabolic pathways. Our study aimed to address the challenge of identifying reliable biomarkers for early detection of bronchopulmonary dysplasia (BPD) in preterm infants using metabolomics techniques. Despite numerous efforts in this area, consistent evidence has remained elusive. Through a systematic review and meta-analysis, we meticulously analyzed differences in small-molecule metabolites between BPD and non-BPD preterm infants. Our comprehensive literature search across major English and Chinese databases minimized the likelihood of overlooking crucial reports. This study represents the first qualitative synthesis to assess the correlation between various metabolites and the development of BPD in preterm infants. Additionally, we provided a thorough description of pertinent metabolic pathways, enhancing the understanding of BPD pathogenesis. Despite significant heterogeneity in most findings, the studies included in our analysis demonstrated high quality.

We believe that our study contributes significantly to the field of neonatology and metabolomics research. The identification of reliable biomarkers for BPD detection in preterm infants is crucial for improving clinical outcomes and guiding therapeutic interventions. We hope that our manuscript will be considered for publication in Italian Journal of Pediatrics.

Thank you for considering our submission. We look forward to your response.

Sincerely,

Yanping Guo
